# 3,6,8-Tribromo-7-ethyl­amino-4-methyl-2*H*-chromen-2-one

**DOI:** 10.1107/S1600536812009221

**Published:** 2012-03-10

**Authors:** Ting Zhang, Hai-tao Xi, Chun-bao Miao, Liang Chen, Xiao-qiang Sun

**Affiliations:** a Key Laboratory of Fine Chemical Engineering, Changzhou University, Changzhou 213164, Jiangsu, People’s Republic China

## Abstract

In the title mol­ecule, C_12_H_10_Br_3_NO_2_, the 2*H*-chromen ring is essentially planar (r.m.s. deviation = 0.022 Å) with the ethyl­amino group oriented at 13.9 (5)° with respect to the ring. The mol­ecular structure is stabilized by intra­molecular N—H⋯Br and C—H⋯Br interactions.

## Related literature

For the synthetic procedure, see: Belluti *et al.* (2010 ▶). For a related structure, see: Kruszynski *et al.* (2005 ▶).
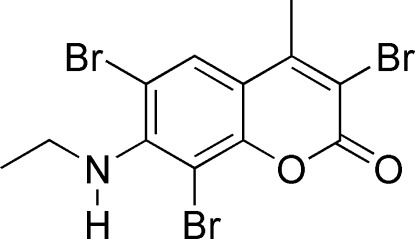


## Experimental

### 

#### Crystal data

C_12_H_10_Br_3_NO_2_
*M* *_r_* = 439.94
Monoclinic, 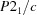

*a* = 8.5045 (9) Å
*b* = 7.2551 (8) Å
*c* = 21.556 (2) Å
β = 94.720 (2)°
*V* = 1325.5 (3) Å^3^
*Z* = 4
Mo *K*α radiation
μ = 9.12 mm^−1^
*T* = 296 K
0.20 × 0.18 × 0.15 mm

#### Data collection

Enraf–Nonius CAD-4 diffractometer
Absorption correction: ψ scan (North *et al.*, 1968 ▶) *T*_min_ = 0.263, *T*_max_ = 0.342
7355 measured reflections
2457 independent reflections
2002 reflections with *I* > 2σ(*I*)
*R*_int_ = 0.034
3 standard reflections every 200 reflections intensity decay: 1%

#### Refinement

*R*[*F* ^2^ > 2σ(*F* ^2^)] = 0.032
*wR*(*F* ^2^) = 0.099
*S* = 1.01
2457 reflections
169 parameters
1 restraint
H atoms treated by a mixture of independent and constrained refinement
Δρ_max_ = 0.63 e Å^−3^
Δρ_min_ = −0.56 e Å^−3^

### 

Data collection: *CAD-4 Software* (Enraf–Nonius, 1985) ▶; cell refinement: *CAD-4 Software* ▶; data reduction: *XCAD4* (Harms & Wocadlo,1995 ▶); program(s) used to solve structure: *SHELXS97* (Sheldrick, 2008 ▶); program(s) used to refine structure: *SHELXL97* (Sheldrick, 2008 ▶); molecular graphics: *SHELXTL* (Sheldrick, 2008 ▶); software used to prepare material for publication: *SHELXTL*.

## Supplementary Material

Crystal structure: contains datablock(s) I, global. DOI: 10.1107/S1600536812009221/pv2515sup1.cif

Structure factors: contains datablock(s) I. DOI: 10.1107/S1600536812009221/pv2515Isup2.hkl

Supplementary material file. DOI: 10.1107/S1600536812009221/pv2515Isup3.cml

Additional supplementary materials:  crystallographic information; 3D view; checkCIF report

## Figures and Tables

**Table 1:** Hydrogen-bond geometry (Å, °)

*D*—H⋯*A*	*D*—H	H⋯*A*	*D*⋯*A*	*D*—H⋯*A*
N1—H1⋯Br1	0.87 (1)	2.64 (4)	3.039 (4)	109 (3)
C10—H10*A*⋯Br2	0.96	2.60	3.176 (5)	118
C13—H13*A*⋯Br3	0.97	2.71	3.146 (7)	108

**Fig. 1.**
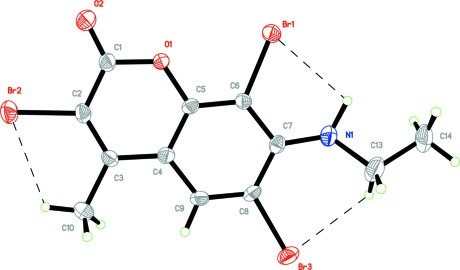
The molecular structure of (I), with the atom-numbering scheme. Displacement ellipsoids are drawn at the 50% probability level.

**Fig. 2.**
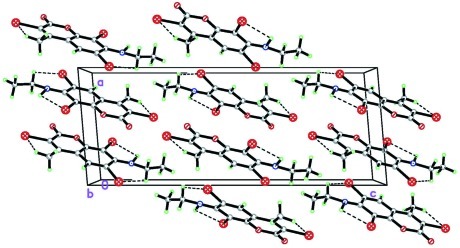
A packing diagram for (I).

## supplementary crystallographic information

### Comment

The title compound is used as an important intermediate in the synthesis of fluorescent tracers (Belluti *et al.*, (2010). The 2*H*-chromen ring in the title molecule (Fig. 1) is essentially planar (rmsd 0.022) with ethylamino group oriented at 13.9 (5)° with respect to the ring. The molecular dimensions of the title compound are in agreement with the corresponding dimensions of the structure of a related compound (Kruszynski *et al.*, 2005).

In the crystal of the title compound, there are only N—H···Br and C—H···Br intramolecular hydrogen bonds which stabilize the molecular structure.

### Experimental

The title compound was prepared by a method reported in the literature (Belluti *et al.*, (2010)). To a suspension of 4-methyl-7-*N*,*N*-diethylamino coumarin (10 mmol, 2.31 g) and bromosuccinimide (11 mmol, 1.95 g) in carbon tetrachloride (100 ml), a catalytic amount of benzoyl peroxide was added. The reaction mixture was refluxed for 8 h, the succinimide thus produced during the reaction was filtered off, and the solvent was washed with water, dried and removed under reduced pressure to afford the title compound as a pale yellow product. Colorless block of the title compound were grown in ethanol (20 ml) by evaporating the solvent slowly at room temperature for about 5 days.

### Refinement

All H atoms were positioned geometrically and constrained to ride on their parent atoms, with C—H = 0.93 Å for aromatic H and 0.86 (1) Å for N—H; with *U*_iso_(H) = *xU*_eq_(C), where *x* = 1.2 for aromatic H, and *x* = 1.5 for other H.

### Crystal data

**Table:**

C_12_H_10_Br_3_NO_2_	*F*(000) = 840
*M**_r_* = 439.94	*D*_x_ = 2.205 Mg m^−^^3^
Monoclinic, *P*2_1_/*c*	Mo *K*α radiation, λ = 0.71073 Å
Hall symbol: -P 2ybc	Cell parameters from 2620 reflections
*a* = 8.5045 (9) Å	θ = 2.4–25.5°
*b* = 7.2551 (8) Å	µ = 9.12 mm^−^^1^
*c* = 21.556 (2) Å	*T* = 296 K
β = 94.720 (2)°	BLOCK, colorless
*V* = 1325.5 (3) Å^3^	0.20 × 0.18 × 0.15 mm
*Z* = 4	

### Data collection

**Table:**

Enraf–Nonius CAD-4 diffractometer	2002 reflections with *I* > 2σ(*I*)
Radiation source: fine-focus sealed tube	*R*_int_ = 0.034
Graphite monochromator	θ_max_ = 25.5°, θ_min_ = 1.9°
ω/2θ scans	*h* = −9→10
Absorption correction: ψ scan (North *et al.*, 1968)	*k* = −8→8
*T*_min_ = 0.263, *T*_max_ = 0.342	*l* = −26→23
7355 measured reflections	3 standard reflections every 200 reflections
2457 independent reflections	intensity decay: 1%

### Refinement

**Table:**

Refinement on *F*^2^	Primary atom site location: structure-invariant direct methods
Least-squares matrix: full	Secondary atom site location: difference Fourier map
*R*[*F*^2^ > 2σ(*F*^2^)] = 0.032	Hydrogen site location: inferred from neighbouring sites
*wR*(*F*^2^) = 0.099	H atoms treated by a mixture of independent and constrained refinement
*S* = 1.01	*w* = 1/[σ^2^(*F*_o_^2^) + (0.0605*P*)^2^ + 0.6207*P*] where *P* = (*F*_o_^2^ + 2*F*_c_^2^)/3
2457 reflections	(Δ/σ)_max_ < 0.001
169 parameters	Δρ_max_ = 0.63 e Å^−^^3^
1 restraint	Δρ_min_ = −0.56 e Å^−^^3^

### Special details

**Table:**

Geometry. All e.s.d.'s (except the e.s.d. in the dihedral angle between two l.s. planes) are estimated using the full covariance matrix. The cell e.s.d.'s are taken into account individually in the estimation of e.s.d.'s in distances, angles and torsion angles; correlations between e.s.d.'s in cell parameters are only used when they are defined by crystal symmetry. An approximate (isotropic) treatment of cell e.s.d.'s is used for estimating e.s.d.'s involving l.s. planes.
Refinement. Refinement of *F*^2^ against ALL reflections. The weighted *R*-factor *wR* and goodness of fit *S* are based on *F*^2^, conventional *R*-factors *R* are based on *F*, with *F* set to zero for negative *F*^2^. The threshold expression of *F*^2^ > σ(*F*^2^) is used only for calculating *R*-factors(gt) *etc*. and is not relevant to the choice of reflections for refinement. *R*-factors based on *F*^2^ are statistically about twice as large as those based on *F*, and *R*- factors based on ALL data will be even larger.

### Fractional atomic coordinates and isotropic or equivalent isotropic displacement parameters (Å^2^)

**Table:**

	*x*	*y*	*z*	*U*_iso_*/*U*_eq_	
Br1	0.35825 (6)	0.11548 (6)	0.05870 (2)	0.04316 (16)	
Br2	0.41792 (7)	0.58281 (7)	−0.22380 (2)	0.05645 (19)	
Br3	0.01975 (6)	0.79144 (7)	0.09690 (2)	0.05198 (18)	
O1	0.3974 (3)	0.3023 (4)	−0.05962 (13)	0.0364 (7)	
C8	0.1457 (5)	0.6305 (6)	0.0522 (2)	0.0354 (9)	
C5	0.3144 (4)	0.4156 (5)	−0.02319 (19)	0.0313 (9)	
C4	0.2630 (5)	0.5889 (5)	−0.0453 (2)	0.0335 (9)	
C7	0.1975 (5)	0.4566 (6)	0.07545 (19)	0.0335 (9)	
C2	0.3720 (5)	0.5264 (6)	−0.14159 (19)	0.0377 (10)	
O2	0.5003 (4)	0.2360 (5)	−0.14659 (15)	0.0560 (9)	
C9	0.1786 (5)	0.6931 (5)	−0.0049 (2)	0.0359 (9)	
H9	0.1434	0.8097	−0.0174	0.043*	
C1	0.4277 (5)	0.3477 (6)	−0.1195 (2)	0.0371 (10)	
C3	0.2947 (5)	0.6444 (5)	−0.1069 (2)	0.0334 (9)	
C6	0.2846 (5)	0.3532 (5)	0.03440 (19)	0.0314 (9)	
N1	0.1665 (5)	0.3812 (6)	0.13117 (19)	0.0511 (11)	
C10	0.2404 (6)	0.8311 (7)	−0.1302 (2)	0.0519 (12)	
H10A	0.2757	0.8515	−0.1708	0.078*	
H10B	0.2836	0.9243	−0.1021	0.078*	
H10C	0.1273	0.8365	−0.1325	0.078*	
C13	0.1232 (8)	0.4655 (9)	0.1875 (3)	0.0650 (15)	
H13A	0.0167	0.5137	0.1812	0.078*	
H13B	0.1938	0.5673	0.1986	0.078*	
C14	0.1322 (8)	0.3266 (9)	0.2388 (2)	0.0668 (15)	
H14A	0.0715	0.2198	0.2258	0.100*	
H14B	0.0906	0.3791	0.2749	0.100*	
H14C	0.2402	0.2916	0.2488	0.100*	
H1	0.218 (6)	0.287 (5)	0.147 (2)	0.064 (18)*	

### Atomic displacement parameters (Å^2^)

**Table:**

	*U*^11^	*U*^22^	*U*^33^	*U*^12^	*U*^13^	*U*^23^
Br1	0.0588 (3)	0.0282 (2)	0.0433 (3)	0.00216 (19)	0.0097 (2)	0.00324 (18)
Br2	0.0814 (4)	0.0539 (3)	0.0367 (3)	0.0003 (3)	0.0208 (3)	0.0071 (2)
Br3	0.0578 (3)	0.0458 (3)	0.0550 (3)	0.0094 (2)	0.0203 (2)	−0.0111 (2)
O1	0.0488 (17)	0.0293 (15)	0.0323 (16)	0.0073 (13)	0.0110 (13)	0.0001 (12)
C8	0.039 (2)	0.033 (2)	0.034 (2)	0.0012 (18)	0.0082 (19)	−0.0109 (17)
C5	0.033 (2)	0.0264 (19)	0.035 (2)	−0.0005 (16)	0.0062 (18)	−0.0065 (17)
C4	0.038 (2)	0.028 (2)	0.035 (2)	−0.0015 (17)	0.0053 (18)	−0.0023 (17)
C7	0.035 (2)	0.036 (2)	0.030 (2)	−0.0066 (18)	0.0046 (17)	−0.0056 (18)
C2	0.049 (2)	0.037 (2)	0.028 (2)	−0.005 (2)	0.0055 (19)	0.0018 (18)
O2	0.083 (3)	0.0467 (19)	0.041 (2)	0.0135 (19)	0.0224 (19)	−0.0055 (16)
C9	0.041 (2)	0.025 (2)	0.042 (3)	0.0028 (17)	0.0050 (19)	−0.0048 (18)
C1	0.048 (2)	0.032 (2)	0.032 (2)	−0.0022 (19)	0.009 (2)	−0.0029 (18)
C3	0.040 (2)	0.027 (2)	0.034 (2)	−0.0009 (17)	0.0014 (18)	0.0025 (17)
C6	0.039 (2)	0.0244 (19)	0.031 (2)	−0.0015 (17)	0.0038 (18)	−0.0006 (16)
N1	0.071 (3)	0.048 (3)	0.037 (2)	0.004 (2)	0.017 (2)	0.0048 (18)
C10	0.065 (3)	0.040 (2)	0.051 (3)	0.015 (2)	0.011 (3)	0.014 (2)
C13	0.086 (4)	0.068 (4)	0.043 (3)	−0.006 (3)	0.020 (3)	−0.009 (3)
C14	0.089 (4)	0.079 (4)	0.034 (3)	−0.011 (3)	0.014 (3)	0.005 (3)

### Geometric parameters (Å, º)

**Table:**

Br1—C6	1.894 (4)	O2—C1	1.200 (5)
Br2—C2	1.891 (4)	C9—H9	0.9300
Br3—C8	1.899 (4)	C3—C10	1.503 (6)
O1—C5	1.372 (5)	N1—C13	1.434 (6)
O1—C1	1.376 (5)	N1—H1	0.866 (10)
C8—C9	1.362 (6)	C10—H10A	0.9600
C8—C7	1.415 (6)	C10—H10B	0.9600
C5—C6	1.365 (6)	C10—H10C	0.9600
C5—C4	1.402 (6)	C13—C14	1.494 (8)
C4—C9	1.396 (6)	C13—H13A	0.9700
C4—C3	1.434 (6)	C13—H13B	0.9700
C7—N1	1.365 (6)	C14—H14A	0.9600
C7—C6	1.415 (6)	C14—H14B	0.9600
C2—C3	1.344 (6)	C14—H14C	0.9600
C2—C1	1.448 (6)		
			
C5—O1—C1	122.5 (3)	C5—C6—C7	122.6 (4)
C9—C8—C7	122.4 (4)	C5—C6—Br1	118.1 (3)
C9—C8—Br3	114.8 (3)	C7—C6—Br1	119.2 (3)
C7—C8—Br3	122.8 (3)	C7—N1—C13	131.0 (4)
C6—C5—O1	117.7 (3)	C7—N1—H1	122 (4)
C6—C5—C4	122.0 (4)	C13—N1—H1	99 (4)
O1—C5—C4	120.3 (4)	C3—C10—H10A	109.5
C9—C4—C5	115.9 (4)	C3—C10—H10B	109.5
C9—C4—C3	124.8 (4)	H10A—C10—H10B	109.5
C5—C4—C3	119.3 (4)	C3—C10—H10C	109.5
N1—C7—C8	126.3 (4)	H10A—C10—H10C	109.5
N1—C7—C6	119.2 (4)	H10B—C10—H10C	109.5
C8—C7—C6	114.5 (4)	N1—C13—C14	109.8 (5)
C3—C2—C1	123.3 (4)	N1—C13—H13A	109.7
C3—C2—Br2	122.2 (3)	C14—C13—H13A	109.7
C1—C2—Br2	114.5 (3)	N1—C13—H13B	109.7
C8—C9—C4	122.5 (4)	C14—C13—H13B	109.7
C8—C9—H9	118.7	H13A—C13—H13B	108.2
C4—C9—H9	118.7	C13—C14—H14A	109.5
O2—C1—O1	116.0 (4)	C13—C14—H14B	109.5
O2—C1—C2	127.7 (4)	H14A—C14—H14B	109.5
O1—C1—C2	116.2 (4)	C13—C14—H14C	109.5
C2—C3—C4	118.3 (4)	H14A—C14—H14C	109.5
C2—C3—C10	122.6 (4)	H14B—C14—H14C	109.5
C4—C3—C10	119.1 (4)		
			
C1—O1—C5—C6	176.9 (4)	C1—C2—C3—C4	−1.4 (6)
C1—O1—C5—C4	−3.0 (6)	Br2—C2—C3—C4	179.1 (3)
C6—C5—C4—C9	−0.4 (6)	C1—C2—C3—C10	178.8 (4)
O1—C5—C4—C9	179.6 (3)	Br2—C2—C3—C10	−0.7 (6)
C6—C5—C4—C3	−178.4 (4)	C9—C4—C3—C2	−177.2 (4)
O1—C5—C4—C3	1.5 (6)	C5—C4—C3—C2	0.6 (6)
C9—C8—C7—N1	−178.1 (4)	C9—C4—C3—C10	2.6 (7)
Br3—C8—C7—N1	−0.1 (6)	C5—C4—C3—C10	−179.5 (4)
C9—C8—C7—C6	−0.5 (6)	O1—C5—C6—C7	−178.7 (3)
Br3—C8—C7—C6	177.5 (3)	C4—C5—C6—C7	1.2 (6)
C7—C8—C9—C4	1.4 (7)	O1—C5—C6—Br1	−0.6 (5)
Br3—C8—C9—C4	−176.8 (3)	C4—C5—C6—Br1	179.3 (3)
C5—C4—C9—C8	−0.9 (6)	N1—C7—C6—C5	177.1 (4)
C3—C4—C9—C8	177.0 (4)	C8—C7—C6—C5	−0.7 (6)
C5—O1—C1—O2	−179.4 (4)	N1—C7—C6—Br1	−1.1 (5)
C5—O1—C1—C2	2.2 (6)	C8—C7—C6—Br1	−178.8 (3)
C3—C2—C1—O2	−178.2 (5)	C8—C7—N1—C13	−22.3 (9)
Br2—C2—C1—O2	1.4 (6)	C6—C7—N1—C13	160.2 (5)
C3—C2—C1—O1	0.0 (6)	C7—N1—C13—C14	−169.4 (5)
Br2—C2—C1—O1	179.5 (3)		

### Hydrogen-bond geometry (Å, º)

**Table:**

*D*—H···*A*	*D*—H	H···*A*	*D*···*A*	*D*—H···*A*
N1—H1···Br1	0.87 (1)	2.64 (4)	3.039 (4)	109 (3)
C10—H10*A*···Br2	0.96	2.60	3.176 (5)	118
C13—H13*A*···Br3	0.97	2.71	3.146 (7)	108
